# Blood safety and epidemiological trends of blood-borne infections in Brazil: A retrospective analysis

**DOI:** 10.1016/j.bjid.2025.104592

**Published:** 2025-10-31

**Authors:** Rodrigo Guimarães Cunha, Elba Regina Sampaio de Lemos, Luiz de Melo Amorim Filho, Maria Esther Duarte Lopes, Marco Aurelio Pereira Horta, Renata Carvalho de Oliveira

**Affiliations:** a Instituto Estadual de Hematologia Arthur de Siqueira Cavalcanti, Rio de Janeiro, RJ, Brazil; b Instituto Oswaldo Cruz/FIOCRUZ, Manguinhos, RJ, Brazil

**Keywords:** Blood donations, Bblood screening, Transfusion-transmissible infection, Blood donor profile, Transfusional safety

## Abstract

The transfusion of blood components is a critical therapeutic intervention for certain clinical conditions for which alternative treatments are often unavailable. Despite the benefits, transfusions can pose health risks to the recipients, including potential transmission of infectious agents. Post-donation, blood components undergo testing for major transmissible agents such as Human Immunodeficiency Virus (HIV), Hepatitis B and C Viruses (HBV and HCV), *Treponema pallidum* (syphilis), and *Trypanosoma cruzi* (Chagas disease). This retrospective study assessed the prevalence of donor unsuitability due to these agents and examined potential influences on the profile of blood-borne infections among healthy blood donors in the region. This study was conducted at a public institution in Brazil from January 2014 to December 2021. All effective blood donations were included, totaling 600,001. The donor demographic profile was as follows: 60.5% male, 52.2% single, 44.5% self-identified as white, 39.6% completed high school, and the 31–40 year age group had the highest number of donors, comprising 28%, with the majority being regular donors (70.4%). The prevalence of hemotransmissible agents was 2.13% (*T. pallidum*), 1.54% (HBV), 0.44% (HIV), 0.36% (*T. cruzi*), 0.26% (HCV), and 0.23% (HTLV). Specific associations were noted among the sociodemographic data for each condition. *T. pallidum* and HBV infections are the most frequent causes of donor ineligibility. The data indicate that prevalence rates remained relatively constant with minor fluctuations throughout the study period, although the frequency of HIV infection was notably higher in 2021.

## Introduction

The transfusion of blood components, including red blood cell concentrates, platelets, plasma, and cryoprecipitates, is a critical treatment for many diseases, and in some situations, it is the only therapy available to patients [1] Despite its substantial benefits, transfusion poses risks of infectious and non-infectious complications to recipients [2] Among infectious complications, the transmission of pathogens such as Human Immunodeficiency Virus (HIV), Hepatitis B and C Viruses (HBV and HCV), *Treponema pallidum*, and *Trypanosoma cruzi*, the protozoan agents of Chagas disease, are particularly noteworthy. The inclusion of other microorganisms in screening protocols is contingent upon regional and local epidemiological factors [2]

According to the World Health Organization (WHO), the risk of infection transmission via blood donation is influenced by the specific infectious agents and the socioeconomic conditions of the country. In countries with high socioeconomic status, the risk of HIV transmission is 0.001%, HBV 0.01%, HCV 0.06%, and syphilis 0.01%. Conversely, in countries with low socioeconomic development, the risk of transmission is HIV 0.7%, HBV 2.81%, HCV 1%, and syphilis 0.92% [2]

In Brazil, the Ministry of Health mandates comprehensive blood screening procedures requiring laboratory testing of all donors for HBV, HCV, HIV, HTLV, *T. cruzi*, and *T. pallidum*, with additional testing for *Plasmodium* spp. in the Amazon region due to malaria risk. Furthermore, clinical and epidemiological histories, including lifestyle factors that may influence transmission risk are assessed [3] Serological screening tests are performed, and since 2014, Nucleic Acid Testing (NAT) for HIV and HCV was implemented, with HBV testing added two years later and *Plasmodium* spp. detection in 2022[3] As a result, NAT is now able to identify HIV, HCV, HBV and *Plasmodium* spp. These procedures facilitate the early detection of infected donors during the immunological window period of serological assays [4]

Given Brazil’s vast geographical expanse and diverse biomes, the prevalence of screening conditions may vary among donors according to the region. Therefore, a study conducted from 2010 to 2016 at the blood center of Goiás State, located in the Cerrado biome, reported a prevalence of 0.21% for HIV, 1.63% for HBV, 0.46% for HCV, 0.87% for syphilis, 0.009% for HTLV, and 0.21% for Chagas disease[5] Another study conducted between 2010 and 2016 in the state of Bahia within the Atlantic Forest biome identified an average prevalence of 0.63% for HIV, 0.65% for hepatitis B (0.51% anti-HBcAg and HBsAg, 0.14%), 0.15% for HCV, 1.09% for syphilis, 0.14% for HTLV, and 0.10% for *T. cruzi* [6]

In addition to the disqualification of blood donor candidates due to various factors, including those associated with transfusion-transmissible infection, many blood centers in Brazil encounter challenges in maintaining adequate levels of blood supplies. Consequently, diverse strategies to increase donor recruitment, such as external collections and mass media campaigns are necessary to fulfill the growing demand. It is also crucial to identify the primary infectious agents causing ineligibility to support local health policies aimed at preventing these diseases. Monitoring the prevalence of transmissible agents in blood donors is essential to increase the safety of transfusions. This study aimed to ascertain the prevalence of infections transmitted through blood components that are part of routine testing in blood donors at a hemocenter in the Southeastern region of Brazil from 2014 to 2021.

## Methods

### Study site

The study was conducted at a central public institution. receiving an average of 7,600 blood donations per month, in addition to providing assistance and support to health services.

### Sampling

The study population comprised all blood donors who successfully donated blood after passing clinical screening. Samples were collected from donors who donated at the blood center as well as during external collections campaigns conducted off-site. The study period covered donations made between 2014 and 2021, totaling 600,001 donors. The infectious diseases analyzed in this study were those subject to mandatory testing in Brazil according to Ministry of Health regulations: HBV, HCV, HIV, HTLV, *T. cruzi* and *T. pallidum*. A database containing socioeconomic information and the following variables was created: sex, age, race, educational level, marital status, and whether it was the donor's initial donation or subsequent donations. All data were retrieved from donor records.

### Donor testing

Blood donors were volunteers who did not receive any form of compensation for their donations. Screening involved completing a specific questionnaire, weighing, and hemoglobin analysis, followed by blood donation upon candidate approval. After donation, blood was submitted for serological laboratory testing for HBV, HCV, HIV, HTLV, *T. cruzi* and *T. pallidum*. Sample analysis was conducted using the following commercial serological tests: (i) HBV (HBsAg EIA/MUREX or CMIA/Abbott) and (Anti-HBcAg CMIA/Diasorin or EIA/Murex3); (ii) HCV-EIA/MUREX or Abbott/CMIA; (iii) HIV ‒ EIA/MUREX or CMIA/Diasorin; and (iv) HTLV – EIA/MUREX or CMIA/Abbott (v) Chagas disease CMIA/Abbott. Molecular analysis was performed using RT-PCR for HBV, HCV, and HIV using the NAT Plus HIV/HBV/HCV/Plasmodium kit (Bio-Manguinhos). If the sample tested positive in two or more tests for the same agent or in two positive NAT tests, the donor's blood was considered deferred and was not used. For a positive result in the first serological test, a new duplicate test was performed based on the regulations established by the Ministry of Health using different methodologies[3] NAT was performed in pools with up to six donors in the same test. A positive result was complemented by an individual analysis of the blood sample, and a positive donor sample was considered deferred.

### Statistical analysis

The association between sociodemographic variables and positivity for HBV, HCV, HIV, HTLV, *T. cruzi* and *T. pallidum* was evaluated using the Chi-Square test. Logistic regression was employed to estimate the association between the parameters tested using the prevalence odds ratio. Prevalence was determined through frequency analysis. Statistical analyses were performed using RStudio Software (version 4.3.0, R Foundation for Statistical Computing, Vienna, Austria). Relationships were considered statistically significant at p<0.05, with a confidence interval of 95%.

### Ethical considerations

The study protocol was approved by the appropriate Ethics Committee (approval n° 5106467) and was conducted in accordance with the Declaration of Helsinki.

## Results

Between January 2014 and December 2021, 600,001 effective blood donations were analyzed. Among the participants, 60.5% were male, 52.2% were single, 44.5% self-identified as white, 39.6% completed high school, and the 31–40 year age group had the highest number of donors, comprising 28%, with the majority being regular donors (70.4%) (Table 1).

**Table 1:** Sociodemographic characteristics of blood donors during the study period (2014–2021).

**Donor Characteristics**	**n (%)**
Sex	
Female	237,147 (39.5)
Male	362,754 (60.5)
Age group (years)	
17–20	6,787 (1.1)
21–30	142,044 (23)
31–40	168,565 (28)
41–50	144,082 (24)
51–65	122,035 (20.3)
>65	16,388 (2.7)
Marital status, n (%)	
Married	248,796 (41.5)
Divorced	31,380 (5.2)
Single	313,202 (52.2)
Widower	5,201 (0.9)
Race, n (%)	
White	266,824 (44.5)
Indigenous	290 (0)
Black	64,622 (10.8)
Eastern	1,867 (0.3)
Pardo	266,266 (44.4)
Scholarity, n (%)	
Unlettered	1,555 (0.3)
Incomplete elementary	42,421 (7.1)
Complete elementary	33,871(5.6)
Incomplete high school	37,484 (6.2)
Complete high school	237,363(39.6)
Incomplete higher education	89,800 (15)
Complete higher education	157,407 (26.2)
First time donation, n (%)	
No	422,243(70.4)
Yes	177,658 (29.6)

Syphilis was the infection with the highest prevalence (2.13%), with the highest prevalence in 2014 (2.42%), and the lowest in 2019 (1.95%). Hepatitis B was the second most prevalent infection at 1.54%, peaking in 2017 (1.78%) and reaching its lowest in 2020 (1.28%). HTLV was the least prevalent infection (0.23%), with the highest prevalence in 2019 (0.43%) and the lowest in 2021 (0.16%). Other infections exhibited the following prevalence rates were identified: (i) *T. cruzi* (0.36%), with the highest prevalence (1.49%) in 2017 and lowest (0.08%) in 2019; (ii) HCV (0.26%), with 0.56% in 2014 and 0.11% in 2019; and (iii) HIV (0.44%), with the highest prevalence in 2021 (0.8%) and lowest in 2019 (0.32%) (Table 2).

**Table 2:** Prevalence of *T. cruzi,* HBV, HCV, HIV, HTLV and *T. pallidum* in blood donors from HEMORIO, Rio de Janeiro state, Brazil 2014–2021.

**Year**	**Blood donors**	***T. cruzi* (%)**	**HBV (%)**	**HCV (%)**	**HIV (%)**	**HTLV (%)**	***T. pallidum* (%)**
2014	66,275	0.09	1.49	0.56	0.35	0.21	2.42
2015	66,400	0.26	1.31	0.32	0.34	0.17	2.07
2016	62,070	0.24	1.55	0.24	0.4	0.21	2.06
2017	80,511	1.49	1.78	0.28	0.41	0.19	2.13
2018	82,629	0.32	1.56	0.19	0.34	0.25	2.01
2019	82,567	0.08	1.6	0.11	0.32	0.43	1.95
2020	78,899	0.13	1.28	0.16	0.54	0.18	2.16
2021	80,650	0.16	1.66	0.3	0.8	0.16	2.24
Total	600,001	2154 (0.36)	9,222 (1.54)	1,582 (0.26)	2,651 (0.44)	1,371 (0.23)	12,754 (2.13)

Among the donors deferred due to *T. cruzi*, there was a higher frequency among males (0.4%), the 21–30 age group (0.5%), single individuals (0.4%), Caucasians (0.4%), those with incomplete higher education (0.5%), and regular donors (0.4%). The analysis revealed a higher chance of being deferred due to *T. cruzi* infection in men (OR = 1.18; p < 0.05) and single individuals (OR = 1.15; p < 0.05).

For donors deferred due to *T. pallidum*, a higher prevalence was observed among males (2.2%), single individuals (5.6%), those over 65-years-old (4.5%), black individuals (3.3%), those without education (5.4%), and first-time donors (5.4%). There was a higher chance of being deferred among male donors (OR = 1.33; p < 0.05), widowed individuals (OR = 1.45; p < 0.05), black individuals (OR = 2.13; p < 0.05), and first-time donors (OR = 2.87; p < 0.05) (Table 3).

**Table 3:** Sociodemographic characteristics of donors and prevalence of diseases transmitted by blood components adjusted by odds ratio (HIV, HTLV and *T. pallidum*).

	**HIV n (%)**	p*	**OR (95% CI)**	**HTLV n (%)**	p*	**OR (95% CI)**	***T. pallidum* n (%)**	p*	**OR (95% CI)**
**Sex**	2,644 (0.4%)	0.98		1,365 (0.2%)	<0.05		12,748 (2.2%)	<0.05	
Female	1,044 (0.4)		1	734 (0.3)		1	4,807 (2)		1
Male	1,600 (0.4)		1.0 (0.92–1.08)	631 (0.2)		0.56 (0.50–0.62)	7,941 (2.2)		**1.33 (1.19‒1.48)**
**Age Group (years)**		<0.05			0.94			<0.05	
17–20	59 (0.9)		**2.75 (1.89–4.0)**	12 (0.2)		0.80 (0.40–1.50)	77 (1.1)		0.24 (0.18–0.3)
21–30	609 (0.4)		**1.35 (1.02–1.81)**	324 (0.2)		1.03 (0.74–1.49)	2,377 (1.7)		0.35 (0.32–0.38)
31–40	791 (0.5)		**1.48 (1.13–1.98)**	378 (0.2)		1.02 (0.73–1.46)	3,267 (1.9)		0.41 (0.38–0.45)
41–50	648 (0.4)		**1.41 (1.08–1.90)**	338 (0.2)		1.06 (0.76–1.53)	2,898 (2.0)		0.43 (0.39–0.46)
51–65	485 (0.4)		1.25 (0.95–1.68)	277 (0.2)		1.03 (0.74–1.48)	3,385 (2.8)		0.59 (0.55–0.65)
> 65	52 (0.3)		1	36 (0.2)		1	744 (4.5)		1
**Marital status**		<0.05			<0.05			<0.05	
Married	972 (0.4)		1	498 (0.2)		1	4,670 (1.9)		1
Separated	121 (0.4)		0.98 (0.81–1.18)	65 (0.2)		1.03 (0.79–1.32)	702 (2.2)		**1.19 (1.10–1.29)**
Single	1,522 (0.5)		**1.24 (1.14–1.35)**	779 (0.2)		**1.24 (1.11–1.39)**	7,221 (5.6)		**1.23 (1.18–1.28)**
Widower	23 (0.4)		1.13 (0.72–1.67)	20 (0.4)		**1.92 (1.18–2.92)**	141 (2.7)		**1.45 (1.22–1.71)**
**Ethnic group**		<0.05			<0.05			<0.05	
White	1,065 (0.4)		1	509 (0.2)		1	4,145 (1.6)		1
Indigenous	2 (0.7)		1.73 (0.28–5.39)	0		–	7 (2.4)		1.56 (0.66–3.08)
Black	358 (0.6)		**1.39 (1.23–1.56)**	154 (0.2)		**1.24 (1.04–1.49)**	2,106 (3.3)		**2.13 (2.02–2.25)**
Oriental	4 (0.2)		0.53 (0.16–1.24)	3 (0.2)		0.84 (0.20–2.19)	31 (1.7)		1.07 (0.73–1.50)
Brown	1,215 (0.5)		**1.14 (1.05–1.24)**	699 (0.3)		**1.37 (1.22–1.54)**	6,458 (2.4)		**1.57 (1.51–1.63)**
**Scholarity**		<0.05			<0.05			<0.05	
Unlettered	11 (0.7)		1	6 (0.4)		1	84 (5.4)		1
Incomplete elementary	216 (0.5)		**0.71 (0.003–0.012)**	154 (0.4)		0.94 (0.45–2.39)	1,842 (4.3)		0.79 (0.63–1.00)
Complete elementary	147 (0.4)		0.61 (0.34–1.20)	74 (0.2)		0.56 (0.26–1.45)	1,177 (3.5)		**0.63 (0.50–0.79)**
Incomplete high school	187 (0.5)		0.70 (0.40–1.37)	115 (0.3)		0.79 (0.38–2.03)	1,189 (3.2)		**0.57 (0.45–0.72)**
Complete high school	1,107 (0.5)		0.65 (0.38–1.27)	581 (0.2)		0.63 (0.31–1.60)	5,523 (2.3)		**0.41 (0.33–0.52)**
Incomplete higher education	414 (0.5)		0.65 (0.37–1.26)	184 (0.2)		0.53 (0.25–1.34)	1,209 (1.3)		**0.23 (0.19–0.30)**
Complete higher education	562 (0.4)		**0.50 (0.29–0.97)**	251 (0.2)		0.41 (0.20–1.04)	1,724 (1.1)		**0.19 (0.15–0.24)**
**First donation**		<0.05			<0.05			<0.05	
No	2,154 (0.5)		1	875 (0.2)		1	5,854 (1.4)		1
Yes	490 (0.3)		**0.53 (0.48–0.59)**	490 (0.3)		**1.33 (1.19–1.48)**	6,894 (5.4)		**2.87 (2.77–2.97)**

Among donors deferred due to HTLV, a higher prevalence was identified among female blood donation candidates (0.3%), widowed (0.4%), mixed-race (0.4%), with incomplete elementary education (0.4%), and first-time donors (0.3%). No age group differences were observed. The association analysis revealed a higher likelihood of deferral among single (OR = 1.24; p < 0.05), widowed (OR = 1.92; p < 0.05), mixed race (OR = 1.37; p < 0.05), Black (OR= 1.24; p<0.05), and first-time donors (OR = 1.33; p < 0.05) (Table 3).

For donors deferred due to HIV infection, there was no difference in prevalence between the sexes (0.4%), with the most prevalent age group being 17‒20 years old (0.9%), with a higher frequency among single individuals (0.5%), those without education (0.7%), and indigenous individuals (0.7%). A higher chance of being deferred was observed among single individuals (OR = 1.24; p < 0.05) and Black individuals (OR = 1.39; p < 0.05). First-time donors and those who completed higher education had a lower chance of being deferred (Table 3).

Among donors deferred due to HBV infection, the distribution between sexes was equal (1.5%), with the most prevalent age group being those over 65-years (2.4%). Among the blood donation candidates, 49.4% were single, 2.3% were black, 3.7% were illiterate, and 2.3% were first-time donors. There was a higher chance of being deferred among widowed (OR = 1.58; p < 0.05), black (OR = 1.90; p < 0.05), and new donors (OR = 1.89; p < 0.05) (Table 4).

**Table 4:** Sociodemographic characteristics of donors and prevalence of diseases transmitted by blood components adjusted by odds ratio (*T. cruzi*, HBV and HCV).

	**Chagas n (%)**	p*	**OR (95% CI)**	**Hepatitis B n (%)**	p*	**OR (95% CI)**	**HepatiTis C n (%)**	p*	**OR (95% CI)**
**Sex**	2,148 (0.4%)	<0.05		9,215 (1.5)	>0.391		1574 (0,3)	0.362	
Female	763 (0.3)		1	3,602 (1.5)		1	604 (0.3)	1	
MaLe	1385 (0.4)		**1.18 (1.08–1.29)**	5,613 (1.5)		1.01 (0.97–1.06)	970 (0.3)		1.04 (0.94–1.16)
**Age Group (years)**		<0.05			<0.05			<0.05	
17–20	5 (0.1)		0.16 (0.05–0.37)	50 (0.7)		**0.3 (0.22–0.27)**	12 (0.2)		**0.27 (0.14–0.48)**
21–30	672 (0.5)		1.09 (0.86–1.40)	1,052 (0.7)		**0.3 (0.26–0.33)**	215 (0.2)		**0.23 (0.18–0.30)**
31–40	510 (0.3)		**0.69 (0.54–0.90)**	2,150 (1.3)		**0.52 (0.46–0.58)**	366 (0.2)		**0.34 (0.27–0.42)**
41–50	469 (0.3)		**0.75 (0.58–0.97)**	2,747 (1.9)		**0.7 (0.70–0.87)**	455 (0.3)		**0.49 (0.40–0.61)**
51–65	421 (0.3)		0.79 (0.62–1.03)	2,821 (2.3)		0.95 (0.86–1.06)	422 (0.3)		**0.54 (0.44–0.67)**
>65	71 (0.4)		1	395 (2.4)		1	104 (0.6)		1
**Marital status**		<0.05			<0.05			<0.05	
Married	834 (0.3%)		1	3,966 (16)		1	688 (0.3)		1
Separated	86 (0.3%)		0.81 (0.64–1.01)	553 (18)		**1.10 (1.01–1.21)**	88 (0.3)		**1.10 (1.01–1.2)**
Single	1217 (0.4%)		**1.15 (1.06–1.26)**	4,549 (49.4)		**0.90 (0.87–0.94)**	763 (0.2)		**0.90 (0.87–0.94)**
Widower	9 (0.3%)		**0.51 (0.24–0.93)**	130 (14)		**1.58 (1.31–1.87)**	29 (0.6)		**1.58 (1.31–1.87)**
**Ethnic group**		<0.05			<0.05			<0.05	
White	1,041 (0.4%)		1	3,215 (1.2)		1	583 (0.2)		1
Indigenous	0		‒	6 (0.1)		‒	1 (0.3)		1.58 (0.089–7.01)
Black	186 (0.3%)		**0.73 (0.62–0.85)**	1,466 (2.3)		**1.90 (1.78–2.02)**	256 (0.4)		**1.81 (1.56–2.10)**
Oriental	3 (0.2%)		0.41	23 (1.2)			3 (0.2)		0.73 (0.18–1.91)
Brown	918 (0.3)		8.83 (8.08 –0.96)	4,505 (1.7)		**1.41 (1.34–1.47)**	730 (0.3)		**1.25 (1.12–1.40)**
**Scholarity**		<0.05			<0.05			<0.05	
Unlettered	3(0.2)		1	57 (3.7)		1	6 (0.4)		1
Incomplete elementary	135 (0.3)		1.65 (0.62–6.70)	1,393 (3.3)		0.89 (0.68–1.18)	253 (0.6)		1.54 (0.75–3.92)
Complete elementary	91 (0.3)		1.39 (0.52–5.67)	789 (2.3)		**0.62 (0.48–0.83)**	128 (0.4)		0.97 (0.47–2.5)
Incomplete high school	157 (0.4)		2.17 (0.82–8.81)	723		**0.51 (0.39–0.68)**	131 (0.3)		0.9 (0.43–2.31)
Complete high school	804 (0.3)		1.75 (0.67–7.08)	723 (1.9)		**0.41 (0.32–0.55)**	634 (0.3)		0.69 (0.33–1.74)
Incomplete higher education	408 (0.5)		2.36 (0.90–9.52)	906 (1.0)		**0.26 (0.20–0.35)**	186 (0.2)		0.53(0.25–1.36)
Complete higher education	550 (0.3)		1.81 (0.69–7.31)	1,614 (1.0)		**0.27 (0.21–0.36)**	236 (0.1)		**0.38(0.18–0.98)**
**First donation**		<0.05			<0.05			<0.05	
No	1,619 (0.4)		1	5,154 (1.2)		1	1,206 (0.3)		1
Yes	529 (0.3)		**0.77 (0.70–0.85)**	4,061 (2.3)		**1.89 (1.81–1.97)**	368 (0.2)		**0.72(0.64–0.81)**

For donors deferred due to HCV, no difference in frequency between the sexes (0.3%) was observed, with the highest prevalence noted in individuals over 65-years of age (0.6%), widowed individuals (0.6%), black individuals (0.4%), those classified as having incomplete elementary education (0.6%), and individuals who had donated more than once (0.3%). A higher chance of being deferred was identified among widowed (OR = 1.58; p < 0.05), separated (OR = 1.10; p < 0.05), and black individuals (OR = 1.81; p < 0.05) (Table 4).

## Discussion

The potential transmission of infectious agents through blood component transfusion remains a persistent concern necessitating consideration based on the donor population profile derived from comprehensive clinical and laboratory screening. Brazil, a country characterized by its vast geographical expanse comprising different biomes, exhibits distinct epidemiological profiles across its regions. For example, testing for *Plasmodium* spp. should be included in the Amazon region, which is endemic for malaria, reinforcing the importance of effective laboratory screening tailored to regional specificity.

The present study analyzed the results of infectious agent screening tests in blood donors over an eight-year period (2014–2021). Blood donation candidate samples were tested according to the guidelines of the Ministry of Health for mandatory screening for HIV, HCV, HTLV, *T. cruzi* and *T. pallidum* [3] Despite the scarcity of publications on the profile of blood donors in Brazil, particularly from blood banks in the northern region of the Amazon rainforest biome, the results obtained in this study align with those identified from other Brazilian blood centers. The majority of donors was single males, below 50-years of age, and had completed high school [5, 6, 7, 8, 9, 10] In studies conducted in the Southern, Southeastern, and Central-Western regions, the majority of donors were white, whereas in the study conducted in the state of Bahia, located in the northeast region, the majority were classified as mixed-race and black candidates, [10] This distribution is consistent with data from the Brazilian Institute of Geography and Statistics in 2022, which indicated that Bahia had the highest percentage of black individuals (22.4%).

Among the blood-borne infections evaluated in blood donation candidates from 2014 to 2021, the prevalence remained stable with minor occasional increases and no discernible upward trend. Notably, during the years 2020 and 2021, despite the COVID-19 pandemic being associated with social isolation, the anticipated reduction in blood donation was not observed at our institution. This contrasts with findings of studies conducted by national authors [11,12] The impact of the COVID-19 pandemic on blood donation patterns, identified in a systematic review and meta-analysis conducted in 2023 demonstrated a global 25% reduction in blood donation, with some regions experiencing a decrease up to 71%. In some regions of Rio de Janeiro, there was an increase in the number of blood donations between 2% and 10% [12]

Regarding the donors deferred in this study, syphilis emerged as the leading cause, similar to the findings of a study conducted in Bahia, in which 1.08% of blood donation candidates tested positive [6] Similarly, research conducted in the state of Goiás, Central-Western region, reported a syphilis-related deferral rate of 0.87%,[5] with an increasing trend in infection prevalence during the study period, reflecting the national epidemiological trend [13] Attie et al. obtained similar results in the state of Paraná, in the Southern region, reporting a 0.67% positivity rate for syphilis in 248,542 individuals subjected to serological screening using chemiluminescence tests, underscoring the risk of transfusion-transmitted syphilis, when relying solely on the venereal disease research laboratory test [14] These findings highlight the critical concern posed by syphilis, a disease without a vaccine, which remains prevalent and is increasing in incidence in Brazil, particularly in Rio de Janeiro, impacting both the blood supply and public health [13]

When analyzing serological ineligibility due to syphilis, we found that the age group with the highest prevalence was individuals over 65-years (4.5%). Brazil is experiencing an exponential growth in its elderly population, and it is estimated that by the end of 2050 more than 30% of the Brazilian population will be over 60-years-old [15] Alongside population aging, there has been an increase in the number of syphilis cases in older people. Between 2010 and 2021, more than 160,000 cases of syphilis were reported in individuals over 50-years of age [15] In this context, it is important to emphasize that most syphilis cases are asymptomatic and information obtained from blood donor assessments can be used to identify patterns of infection occurrence in low-risk populations [16]

A study conducted in the Brazilian population from 2010 to 2020 demonstrated an increase in syphilis cases in older adults, with the highest incidence observed in males, individuals of white or black race, those with low education, and those aged 60–69 years age group. The South and Southeast regions showed the highest proportions of older people with syphilis, and the cumulative prevalence was 12.84 cases per 100,000 individuals over 60-years [17] Another study conducted in the Federal District reported a high prevalence of syphilis among individuals over 60-years, reaching 12.9% [18] Additionally, a study among blood donors in Paraná between 2015 and 2020 showed that syphilis became the leading cause of serological ineligibility from 2020 onward (0.82%), surpassing hepatitis B, which had been the main cause of serological ineligibility among donors in 2015. The same study also demonstrated a 437% increase in syphilis cases among blood donors over 60-years during the study period [19]

Among older adults, those at greatest risk of syphilis were individuals with low education, male sex (particularly men who have sex with men), the presence of coinfections (especially HIV), and belonging to the pardo (mixed race), black, or indigenous ethnic groups [20] Key factors contributing to vulnerability were inadequate knowledge about Sexually Transmitted Infection (STI) transmission and prevention, societal and healthcare-related prejudice when addressing sexuality in older age, insufficient educational campaigns, gaps in epidemiological surveillance of syphilis among the elderly, lack of awareness about syphilis, inconsistent condom use, physiological changes in the genital tract facilitating STI acquisition, and the use of medications for erectile dysfunction [15, 16, 17, 18, 19, 20] In particular, the lack of regular condom use among older adults further increases the risk of syphilis and other STIs, with surveys indicating that only 16.6% of older individuals use condoms consistently [20]

The second leading cause of serological deferral observed in this study was HBV infection, which was identified as the most frequent cause in another study conducted in the Central-Western region but with a decreasing trend in its prevalence during the investigated period. As hepatitis B is a preventable disease, efforts are required to reduce deferral. Brazil’s endemicity is classified as intermediate to low; however, the Northern region, characterized by a high prevalence of HBsAg, and the highest number of hepatitis delta cases, reinforces the need for focused evaluation of regional population profiles and specificities. Vivadini et al.’s spatial analysis of HBV distribution patterns in Brazil confirmed a high concentration of cases in the northern states within the Amazon rainforest biome [21,22]

The prevalence of hepatitis C was consistent with the findings of a study conducted in the state of Goiás, Central-Western region, where 0.46% of donors were deferred due to HCV, unlike the results obtained in Bahia, with a lower prevalence of 0.15%, and in the state of Pará, in the Amazon region, with a prevalence of 0.07% [5,8]

In contrast to what was identified in the states of Goiás and Pará, the prevalence of HIV-positive samples in this study was higher, similar to that observed in the study in Bahia,[6,10] with an increasing trend during the pandemic period in 2020 and 2021, when prevalence rates of 0.54% and 0.80%, respectively, were observed. These results are consistent with the spatial distribution of acquired immune deficiency syndrome incidence rates in Brazil, where the lowest coefficients were found in the Northern and Central-Western regions [23] In cases of deferral due to HIV infection, we identified that younger age groups exhibited a higher frequency, with the 17–20 year age group demonstrating a likelihood twice as high as other age groups. This evidence raises concerns regarding the possibility of HIV infection among younger individuals.

The overall prevalence of *T. cruzi*-positive donor samples during the study period was 0.36%, higher than those identified in the states of Bahia (0.21%) and Goiás (0.10%), as well as Pará, a state located in the Amazon region, where from 2016 to 2021, the prevalence of anti-*T. cruzi* antibodies was 0.1% [24] Considering that the estimated prevalence of *T. cruzi* infection through blood transfusion in Brazil is 0.21%, the prevalence of 1.49% identified in 2017 is notable [25] The migration of individuals with chronic Chagas disease who apply to donate blood when infected may explain this high prevalence, particularly in the year of the greatest public health crisis in the history of Rio de Janeiro. Another explanation for this pattern of *T. cruzi* infection occurrence compared with other studies is the possibility of false-positive results. When analyzing the prevalence of Chagas disease across age groups, it is evident that seropositive donors were identified in all age groups, a finding that differs from other studies in which positive samples were predominantly observed among donors of older age groups [26,27] Based on this, it is possible to speculate about the occurrence of false positivity. Serological screening for Chagas disease, a neglected tropical disease, is performed using serological methods. When a donor presents a reactive test, a second test is performed with the same sample and by the same methodology (duplicate testing). If both results are reactive, the donor is considered ineligible and referred to a specialized center for follow-up, in order to exclude the possibility of a false-positive result. In our study, it was not possible to access the results of the serological tests performed with different methodologies in donors deemed ineligible due to Chagas disease; therefore, the possibility of false-positive results for this disease cannot be ruled out.

Regarding the research on HTLV infection, although it was not possible to confirm positive samples by western blot or molecular analysis in this study, the results obtained with the analysis using two different serological tests enabled all reactive samples to be discarded, even considering the possibility of false-positive results. The prevalence of 0.23% was similar to that in the study conducted in Goiás, but higher than that identified in the state of Amazonas, where out of 87,402 donors tested, 116 (0.13%) were seroreactive. In the same study, testing of the second sample from these 116 positive donors confirmed reactivity in 41 samples, of which only 24 were confirmed by western blotting. Based on the results published by Morais et al.,[28] it can be concluded that the percentage of HTLV-reactive samples from blood donation candidates in the present study could be classified as suitable for transfusion. According to the Brazilian Ministry of Health, the prevalence rates vary from 0.03% in Santa Catarina in the Southern region to 0.48% in Bahia, with the highest rates found in the Northern and Northeastern regions [29]

Both public and private blood centers in Brazil encounter major challenges in maintaining adequate blood-component stocks to prevent shortages. In this study, it was observed that even during the pandemic, the donation profile at our facility remained stable despite the expected reduction, with some regions reporting reductions up to 71% [11,12]

The introduction of the NAT platform for detecting hepatitis B and C viruses, HIV and *Plasmodium* has been a significant advancement in ensuring the quality of blood products, contributing to increased transfusion safety. However, it will be necessary to expand this procedure to include other infectious agents that can be transmitted via blood transfusions [4]

Regarding donor deferral, the regulations set forth by the Brazilian Ministry of Health on haemotherapeutic procedures underscore the significant prevalence of infectious agents, particularly HBV, considering its preventable nature. The high prevalence of HBV infection in the Amazon region exacerbates the risk of hepatitis delta. Research has identified infectious agents unique to specific biomes, such as *Mansonella ozzardi* filarial nematodes, prevalent in a hyperendemic region in the interior of the Amazonas. Given these regional specificities, it is crucial to consider the possibility of screening for infectious agent’s endemic to different regions that are capable of transmission through blood and blood derivatives, thereby posing a risk to immunocompetent and non-immunocompetent recipients [30]

In light of this study’s findings, it is imperative not only to encourage and promote blood donation, but also to consider the eco-epidemiological profile. This involves testing for infectious agents mandated by current legislation as well as pathogens endemic to different regions of Brazil that could potentially be transmitted by an infected donor via blood and its derivatives.

## Conflicts of interest

The authors declare no conflicts of interest.
